# Dendrimer-Entrapped Gold Nanoparticles as Potential CT Contrast Agents for Localizing Sentinel Lymph Node via Indirect CT Lymphography on Rabbit Model

**DOI:** 10.1155/2018/1230151

**Published:** 2018-04-17

**Authors:** Fang Shi, Yue Yang, Jian Chen, Yan Sha, Yilai Shu, Haitao Wu

**Affiliations:** ^1^Department of Otolaryngology-Head and Neck Surgery, Eye, Ear, Nose and Throat Hospital, Fudan University, Shanghai, China; ^2^Key Clinical Disciplines of Otorhinolaryngology of Shanghai, Shanghai, China

## Abstract

**Objective:**

To investigate the potential use of indirect computed tomography lymphography (CT-LG) with dendrimer-entrapped gold nanoparticles (Au DENPs) in the localization and enhanced imaging of cervical sentinel lymph node (SLN) on rabbit model.

**Methods:**

Twelve rabbits were randomly divided into two groups: the positive control group and the experimental group. In the control group, indirect CT-LG was performed with the injection of 0.5 ml activated carbon nanoparticles (ACNP) and Omnipaque mixture suspension in the right tongue submucosa. CT images were acquired before the injection and 1, 5, 10, and 15 min after the injection, respectively. In the experimental group, indirect CT-LG injection with 0.5 ml Au DENPs suspension was performed in the right tongue submucosa. CT images were obtained before the injection and 1, 5, 10, and 15 min and 1, 2, 6, 24, 48, and 72 h after the injection, respectively. Then, SLN identification and enhancement characteristics were evaluated.

**Results:**

Indirect CT-LG revealed the enhancement of one right deep cervical lymph nodes in all animals, which was SLN. SLN location was marked with black color (ACNP dye) or purple-black color (Au DENPs dye). At each detection time point, the enhanced SLN attenuation values of control rabbits were statistically significantly higher than that of the plain scan, respectively (*P* < 0.05). Also the values of experimental rabbits were statistically significantly higher than that of the control at the same time point after injection (*P* < 0.05). The detection rate of SLN was 100%.

**Conclusions:**

Indirect CT-LG with injection of Au DENPs as CT contrast agents can locate the SLN for a long period of time and enrich the SLN black dye. It is helpful for SLNs identification during the operation.

## 1. Introduction

Cervical lymph node metastases are often noted in squamous cell carcinoma of the head and neck, such as tongue, supraglottic, and hypopharyngeal cancers. The occult metastasis rate for these lymph nodes is as high as 50% [1]. The accurate diagnosis of the cervical lymph node metastases is crucial for carcinoma staging, therapy, and prognosis. Sentinel lymph node (SLN) is the first draining lymph node or group of lymph nodes that is reached by metastatic tumor cells from a primary cancer lesion [2], and SLN status as an important marker could reflect the status of the whole group of drainage lymph nodes. The suspected patients could undergo invasive sentinel lymph node biopsy (SLNB) to have their metastases status staged accurately [3]. At present, SLNB often used blue dye, activated carbon nanoparticles (ACNP) tracing dyes, and radionuclide tracer techniques alone or two in combination. However, SLNB would result in seroma formation, lymphedema, sensory nerve injury, and limitation in the range of motion [4]. Moreover, blue-dye tracer and activated carbon nanoparticle (ACNP) tracing dyes would stain surrounding tissues and could not locate SLNs preoperatively. The radionuclide tracer technique with lymphoscintigraphy is confined to intrinsically poor spatial resolution for imaging anatomic details. In addition, it features radioactive contamination and is only available in nuclear medicine department. Current noninvasive methods to identify SLNs have been clinically used to obtain imaging pictures and mapping location of SLN* in vivo*, for example, magnetic resonance imaging (MRI), computed tomography (CT) [5, 6], ultrasonography, and positron emission tomography (PET) combined with CT. Although it is relatively easy to diagnose metastasis of an enlarged lymph node with enhanced CT or MRI [7–9], clinically negative lymph nodes (cN0) with normal size may harbor occult metastases and it is difficult to separate occult metastasis from nonmetastatic lymph nodes [3]. The use of ultrasonography in lymph node assessment is limited by anatomical location and even when ultrasonography is used together with fine-needle aspiration biopsy (FNAB), its sensitivity varies depending on operator experience and on whether sampling error has been introduced. The clinical potential of PET combined with CT is promising, but that may be restricted by a combination of limited sensitivity for small metastatic deposits and a relatively large number of false positive findings in the node-negative neck. The diagnostic roles of the various available noninvasive lymphatic imaging modalities remain to be determined. Therefore, developing authentic noninvasive techniques that exactly identify lymph node metastasis in cancer patients is attracting great attention from researchers.

Indirect lymphography (LG) is a substitutable technique for SLN identification. Indirect LG combined with CT offers the advantages of superior selective enhancement and high sensitivity and requires low doses of contrast agent [10] and the only adverse side effect is temporary swelling at the injection site [11]. Therefore, indirect CT-LG with a water-soluble nonionic monometric contrast agent has been more and more widely used for identifying SLN by mapping and visualizing SLNs in breast [12], esophageal [13], lung [14], and cutaneous cancers [15] during the last decade, but it has few been applied to cancers of the head and neck [16, 17], on account of the drawbacks of contrast agents. The most commonly used CT imaging agents in clinics are iodinated small molecules such as Omnipaque. But these agents have some shortcomings such as short imaging time and nonspecificity [10, 18, 19]. To overcome these drawbacks, various nanoparticle- (NP-) based contrast agents for CT imaging have been designed and have received considerable attention in recent years, just as it is in the case of nanocarbon particles and gold nanoparticles (Au NPs) [20, 21].

Au NPs as CT contrast agents for blood pool imaging and tumor imaging have been well studied for their many advantages, such as long-term enhancement imaging at the animal models via intravenous injection, good water solubility, colloidal stability, and biocompatibility [22, 23]. These advantages become particularly apparent when CT is used to diagnose tumors diseases in cervix [24], liver [25], and prostate [26] cancers in animal models via intravenous injection, but few studies have focused on the application of Au NPs in the head and neck region. Besides, Au NPs are easily uptaken by phagocytes and gathered in lymph nodes, which may enable Au NPs a targeted contrast agent for the lymph nodes imaging and extend the duration theoretically. Therefore, Au NPs are potentially excellent CT contrast agents for indirect LG imaging to identify SLNs in the head and neck. There have been few studies of indirect LG using Au NPs as CT contrast [27] and it identified the enhanced SLNs 100% in all rabbits by indirect CT-LG. But it did not have controlled trials with Omnipaque and lymphatic tracer.

In order to prove this hypothesis, we use dendrimer-entrapped gold nanoparticles [(Au^0^)_300_-G5.NHAc-*m*PEG_20_] DENPs (Au DENPs), compared with the mixture of ACNP and Omnipaque as a positive control for indirect LG-CT to assess its effectiveness in localizing and enhanced imaging of cervical SLN by dynamically observing the color change and enhancement effect of SLNs.

## 2. Materials and Methods

### 2.1. Animal Model

As New Zealand rabbit has similar anatomical structures of cervical lymph nodes to human, we therefore chose healthy New Zealand rabbits as our animal model for CT scan. ACNP has the ability of targeting lymph nodes and the mixture of Omnipaque (volume ratio = 1 : 1) has similar imaging effects to pure Omnipaque.

The study was approved by the animal care and use committee of Fudan University.

Twelve New Zealand White rabbits each weighing 2.0–2.5 kg served as the animal model. Each rabbit was anaesthetized with an intramuscular injection of ketamine hydrochloride (60 mg/kg) and xylazine hydrochloride (0.5 ml/kg) body weight, while positioned supine on a specially designed operating table with arms and legs stretched fully and symmetrically and with neck extended. After that 6 rabbits in the experimental group were injected Au DENPs (0.5 mL, [Au] = 0.1 m) gently into the right ventrolateral submucosa of the tongue and the other 6 rabbits of the positive control group were injected the mixture of Omnipaque and the nanocarbon particle (0.5 mL, volume ratio = 1 : 1) as the experimental group. The injections were performed within 1 minute each.

### 2.2. Indirect Computed Tomography Lymphography

Animals were imaged on a CT table in the supine position with the neck extended. Transverse CT scanning was performed using a Siemens Somatom Sensation 10 scanner (Siemens, Forchhein, Bavaria, Germany) operated at 120 kV and 150 mAs with a 7–12 cm field of view and a 512 × 512 matrix.

Precontrast CT images were taken to eliminate the possibility of lymph node calcification. Contiguous 1.5 mm thick CT images from the level of the tongue to the sternum were taken 1, 5, 10, 15, and 20 minutes after the injection in the control group and 1, 5, 10, 15, and 20 minutes and 1, 2, 4, 6, 24, 48, and 72 hours after the injection in the experiment group. The enhanced lymph nodes were identified and their locations (corresponding to their position on CT-LG) were marked on the skin with a painting pen.

### 2.3. Statistical Analysis

All the data were displayed in the form of Means ± SD with SPSS version 12.0 software. Paired *t*-test was performed to compare differences in CT attenuation values between experiment group and positive control group at each time. And one-way ANOVA was adopted to compare differences in CT attenuation values within each group. A significance level of *α* = 0.05 was used.

## 3. Results

**Figure 1:**
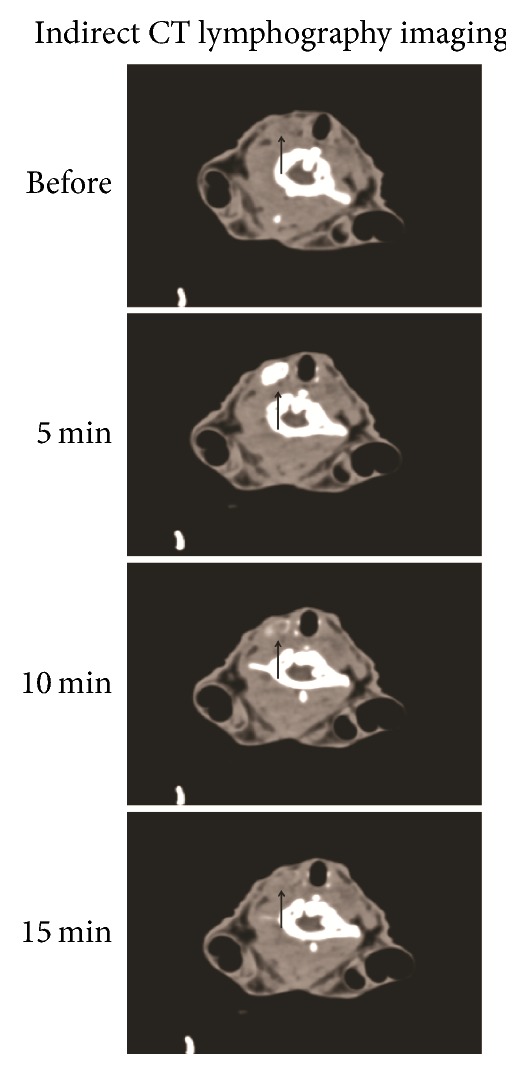
The CT imaging of SLN (black arrow, deep cervical lymph node) before the injection and 5, 10, and 15 minutes after injection of the mixture of Omnipaque and the nanocarbon particle (0.5 mL, volume ratio = 1 : 1) into the right ventrolateral submucosa of the tongue.

Only one right deep cervical lymph node was shown to be enhanced on indirect CT-LG in all rabbits of experimental group and positive control group. SLNs were enhanced homogeneously, and the enhanced SLN in each side of the neck was oval or round in shape with clear boundary and with no filling defect visualized (Figures 1 and 3).

In the positive control group, CT value of sentinel lymph node sites was 59 ± 6.42 HU in the precontrast scan, and it was not statistically significantly different from the value of 20 minutes after injection. CT values of lymph node sites, 1, 5, 10, and 15 minutes after injection of the mixture of Omnipaque and the nanocarbon particle, were 58.8 ± 4.87, 134.8 ± 25.86, 91 ± 11.7, and 63.8 ± 3.87 HU, respectively, which were statistically significantly higher than that of the plain scan, respectively (*P* < 0.05), except the 1- and 15-minute time point. The average diameter of sentinel lymph nodes in the largest cross section was 0.533 ± 0.05 cm in CT and 0.541 ± 0.08 cm in direct anatomy measurement. There were no statistically significant differences between them (*P* > 0.05).

In the experimental group, CT value of sentinel lymph node sites was 44.8 ± 1.7 HU in the precontrast scan. CT values of lymph node sites 1, 5, 10, 15, and 20 min and 1, 2, 6, 24, 48, and 72 h after injection with Au DENPs were 43.6 ± 3.01, 139 ± 16.7, 193.6 ± 7.5, 198.2 ± 8.93, 208.4 ± 9.13, 214.4 ± 14.72, 211.6 ± 7.63, 228 ± 13.02, 198.2 ± 7.78, 176.9 ± 15.22, and 154.0 ± 13.27 HU, respectively. The average diameter of sentinel lymph nodes in the largest cross section was 0.469 ± 0.051 cm in CT and 0.497 ± 0.034 cm in direct anatomy measurement. There were no statistically significant differences between them (*P* > 0.05). At the same time point, the CT value of sentinel lymph nodes in the experimental group was much higher than that in the positive control group. There was statistically significant difference between them (*P* < 0.05) (Figure 5).

**Figure 2:**
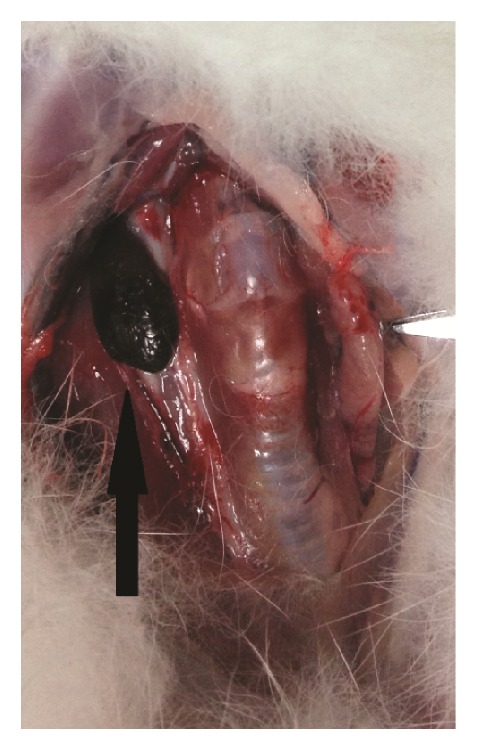
The anatomical observation of SLN (black arrow, deep cervical lymph node) 15 minutes after injection of the mixture of Omnipaque and the nanocarbon particle (0.5 mL, volume ratio = 1 : 1) into the right ventrolateral submucosa of the tongue.

**Figure 3:**
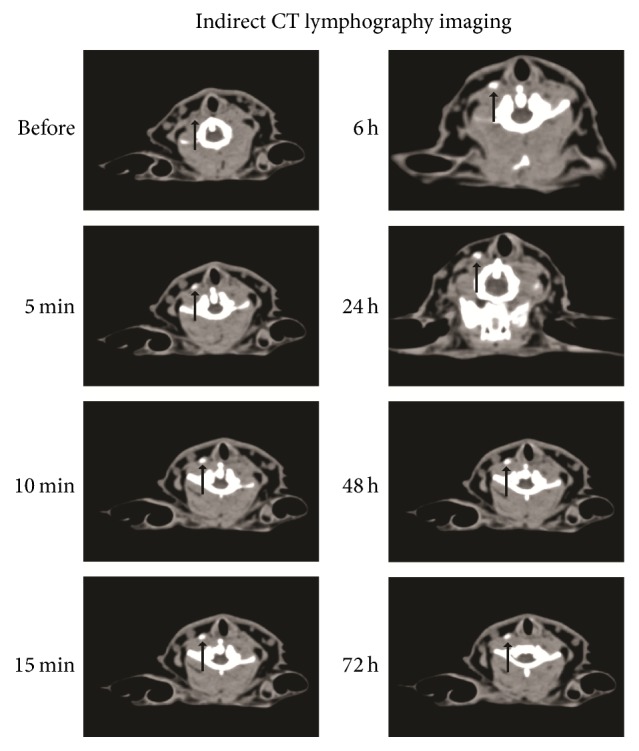
The CT imaging of SLN (black arrow, deep cervical lymph node) before the injection and 5, 10, and 15 minutes and 6, 24, 48, and 72 hours after injection of Au DENPs (0.5 mL, [Au] = 0.1 m) into the right ventrolateral submucosa of the tongue.

**Figure 4:**
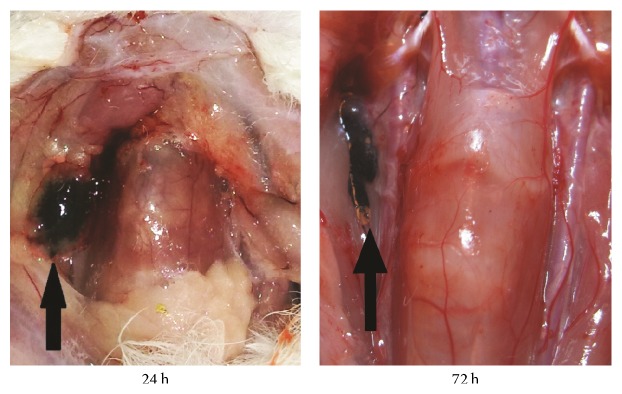
The anatomical observation of SLN (black arrow, deep cervical lymph node) 24 h and 72 h after injection of Au DENPs (0.5 mL, [Au] = 0.1 m) into the right ventrolateral submucosa of the tongue.

**Figure 5:**
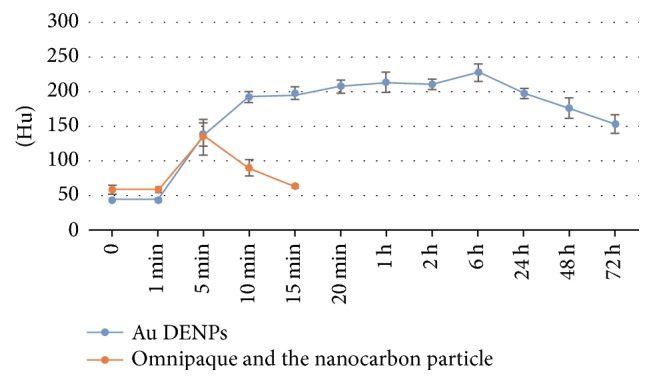
The comparison of CT attenuation values of CT-LG before and after the injection of the mixture of Omnipaque and the nanocarbon particle and Au DENPs at different time points (*n* = 6).

After the cervical anatomy, all of the right deep cervical lymph nodes showed a distinct enrichment of black dye, which was highly correlated with the locations on the indirect CT-LG images (Figures 2 and 4).

## 4. Discussion

At present, indirect CT-LG could be used to identify sentinel lymph nodes as has been reported in various animal models (e.g., rabbit tongue and pyriform sinus VX2 carcinoma) [28, 29] and human patients (e.g., breast, esophageal, cutaneous, and lung cancer patients) [12, 13, 15, 20, 30, 31]. But, Omnipaque, as a common contrast agent for indirect CT-LG that has a short duration of enhancement imaging, would be totally cleared by circulation 15 minutes after injection as has been shown by Figure 1, which was in line with previous studies [28, 32]. Therefore, few clinical applications for SLN identification and biopsy in the deep viscera (e.g., Larynx and hypopharynx) by CT-LG with clinically used iodine containing contrast agents were reported. Thus, the time demands for imaging was pressed from injection in the operation room to scan in CT room and not practical during surgical procedures. Traditionally, SLNs were located preoperatively with indirect CT-LG with iodine agents. However, the SLNs could not be stained intraoperatively, and then clinicians needed to search them carefully in the operation. If lymph nodes could be stained, they would be easily identified, which would increase the detection rate of SLNs. As a good marker for SLNs during surgery through black dye, nanocarbon particle tracing is easy and safe to perform, but it could not be shown on CT images, making it impossible for surgeons to locate the SLNs before surgery. Indirect CT-LG combined with nanocarbon injection was usually performed for easier and more precise identification of SLNs, but injections of different agents were required to be done before and during the operation, respectively. Au NPs, as new molecular agents for X-ray/CT systems imaging in animal models, have been well studied for identification of different kinds of tumors [22, 23, 33, 34]. However, as we have known, there were few studies of indirect CT-LG using Au NPs to identify SLNs of the head and neck.

In the control groups of our study, we used indirect CT-LG with the mixture of Omnipaque and the nanocarbon particle as contrast agent to locate SLNs before surgery and mark them during or after surgery. Only one enhanced SLN, that was all in the same position, lateral to the larynx-trachea region, was visualized through a distinct enrichment of black dye in the ipsilateral side of the neck on CT-LG images after injection of the mixture of Omnipaque and the nanocarbon particle into the right ventrolateral submucosa of the tongue. The actual position of the particle-stained SLNs at dissection was consistent with the position indicated using CT-LG, suggesting that indirect CT-LG could locate the black-dyed SLN before surgery. The mixture of Omnipaque and the nanocarbon particle (ratio of 1 : 1) could not only be used as contrast for CT-LG before surgery, which has almost the same effect as Omnipaque alone, but also help to locate the black-dyed SLNs just as methylene blue [28].

In the experimental groups that were injected with Au DENPs, as we presumed, the deep cervical lymph nodes were enhanced up to 24 hours after injection. This might be attributed to prolonged circulation time due to phagocytosis by macrophage within the lymph node. In consequence, compared with conventional iodine-based agents [28, 32, 35, 36] and the mixture of Omnipaque and the nanocarbon particle, Au DENPs help to yield better CT images and longer enhancement time. As our results show, Au DENPs have a higher X-ray attenuation intensity than the mixture of Omnipaque and the nanocarbon particle, thus making it a promising potential contrast which may be more effective than Omnipaque. After CT scanning, the only one enhanced SLN could also be visualized lateral to the larynx-trachea region through a distinct enrichment of purple-black dye in the ipsilateral side of the neck on CT-LG images, thus making it capable of locating the SLNs quickly and accurately during surgery just as effective as methylene blue and nanocarbon particle. Au DENPs could not only enhance SLNs for more than 24 hours but also stain them, which helps doctors both locate SLNs through CT-LG before surgery and find them quickly and accurately during surgery with just one injection. There is a longer period of time for enhanced imaging of the sentinel lymph nodes before operations via indirect CT-LG combined with injection of Au DENPs as CT contrast agents, which is helpful in finding out SLNs quickly and accurately by making the enrichment of black dye during operations.

In a word, after one injection of Au DENPs under the tongue submucosa, the SLN was identified preoperatively by indirect CT-LG and by the black dye intraoperatively, and the detection rate of lingual SLN was 100%.

## 5. Conclusion

In our present study, the results demonstrated that Au DENPs had characteristics of a well lymphatic system targeting property, exceedingly fast tracing speed, long-time enhancement imaging, and black-dyed lymph node, which were beneficial for SLNs identification preoperatively and intraoperatively. Therefore, we confirmed that Au DENPs could be used as CT contrast agents for SLN identification of indirect CT-LG, and the capacity of long-time enhancement imaging of Au DENPs was superior to conventional agents. In addition, Au DENPs could stain SLNs black, which were helpful for SLNs identification during the operation.
